# Management of Congenital Palatal Fistula Associated With Wiskott-Aldrich Syndrome

**DOI:** 10.7759/cureus.88137

**Published:** 2025-07-17

**Authors:** Teruyuki Niimi, Nagato Natsume, Hideto Imura, Toko Hayakawa, Hiroo Furukawa

**Affiliations:** 1 Division of Research and Treatment for Oral Maxillofacial Congenital Anomalies, School of Dentistry, Aichi Gakuin University, Nagoya, JPN; 2 Cleft Lip and Palate Center, Aichi Gakuin University Dental Hospital, Nagoya, JPN; 3 Division of Research and Treatment for Oral and Maxillofacial Congenital Anomalies, School of Dentistry, Aichi Gakuin University, Nagoya, JPN; 4 Division of Speech, Hearing, and Language, Aichi Gakuin University Dental Hospital, Nagoya, JPN

**Keywords:** congenital palatal fistula, management, oral surgery, submucous cleft palate, wiskott-aldrich syndrome

## Abstract

Submucous cleft palate (SMCP) is a rare form of cleft palate involving abnormal muscle development in the soft palate. Diagnosis is often delayed, with many cases identified after age 4. Symptoms like hypernasality and velopharyngeal insufficiency (VPI) may appear, though some remain asymptomatic. SMCP diagnosis typically relies on Calnan’s triad: bifid uvula, hard palate notch, and zona pellucida. Wiskott-Aldrich syndrome (WAS) is a rare X-linked immune disorder marked by thrombocytopenia, eczema, and infections. Early diagnosis is key to effective treatment. Due to the rarity of both SMCP and WAS, we present a unique case and suggest careful management for optimal outcomes. A one-year and 10-month-old boy with WAS was referred to our cleft center for evaluation of a congenital palatal fistula. There was no history of trauma or surgery, but a 9 × 7 mm midline fistula was observed, along with a diagnosis of SMCP. After hematopoietic stem cell transplantation (HSCT) and continued immunosuppressive therapy, a palatal plate was created to assist feeding. At age 5, a cleft palate repair using a modified Bardach technique was performed. Postoperative healing was successful without complications. This case highlights multidisciplinary management in a patient with both SMCP and WAS.

## Introduction

The submucous cleft palate (SMCP) is a subtype of overt cleft palate, characterized by an innate malformation associated with irregular development in the soft palate's muscle tissue. It is a relatively rare variation of the cleft palate, but is commonly detected through visible features.

In 1931, the first case of a congenital perforation of the hard palate was described by Veau and Borel. In 1954, Calnan presented a triad of clinical criteria to diagnose SMCP, including the bifid uvula, a notch in the posterior end of the hard palate, and palatal muscle diastasis resulting in zona pellucida [1]. Besides that, the term *occult SMCP* was described by Kaplan for patients with velar dysfunction in the absence of one or more anatomical signs of the classic clinical triad [2].

Determining the true prevalence of SMCP is challenging. In the general population worldwide, its incidence is reported to be between 0.02% and 0.08% [3]; however, the accuracy of these figures remains questionable due to potential underestimation from misdiagnosis and underreporting. Asymptomatic SMCP accounts for approximately 50% of all cases, regardless of type [4]. While the diagnosis of cleft palate with cleft lip is straightforward during the newborn period, 25% of all cleft palate cases are diagnosed after 12 months of age [5]. On the other hand, SMCP is typically diagnosed later. Although the average age of detection for asymptomatic SMCP is 4.9 years, some patients may not be diagnosed until eight years of age or older [4]. Recently, ten Dam et al. found that the age of diagnosis for SMCP is 3.7 years [6]. Patients could exhibit symptoms at any age or remain asymptomatic throughout their lives, and it depends on the presence of velopharyngeal insufficiency (VPI). The diagnosis is commonly not recognized unless the children present with symptomatic SMCP characterized by VPI. Individuals with symptomatic SMCP and VPI exhibit an oral-to-nasal resonance imbalance similar to that seen in patients with cleft palate. These symptoms, including hypernasality, nasal air emission, maladaptive articulation errors, middle ear dysfunction, and nasal regurgitation, can lead to feeding problems. Fifty percent of patients with SMCP experience hypernasal speech, making hypernasality the most common symptom of VPI [4]. 

Wiskott-Aldrich syndrome (WAS), a rare X-linked primary immunodeficiency syndrome, is characterized by the clinical triad of microthrombocytopenia (a reduced ability to form blood clots), severe eczema (abnormal patches of red, a form of inflammatory skin disorder), and recurrent infection (immune deficiency). The estimated prevalence of WAS is 1 to 10 per million children in the population, and the disease is recognized, especially in males [7]. The actual incidence may be higher due to the misdiagnosis of mild cases as idiopathic thrombocytopenic purpura.

A prerequisite of WAS treatment is early diagnosis and regular monitoring to improve outcomes, particularly among children without a family history or with milder phenotypes. The management of WAS depends on several factors, notably intensive care, including broad-spectrum antibiotics for bacterial infections, viral and fungal infections. Because of the scarcity of congenital perforation in SMCP and WAS determination, we present an infrequent case and propose a reliable management to reach the optimal outcome for the patient.

## Case presentation

A one-year and 10-month-old boy was referred to the Cleft Lip and Palate Center of our hospital with a chief complaint of a *hole* in the palate. There was no history of palatal trauma or chemical injury, and it was evident that the fistula had been present since birth. The parents confirmed that he had not undergone any surgeries involving the oral region. There was no reported family history of cleft lip or palate.

Since the patient was diagnosed with WAS after birth, hematopoietic stem cell transplantation (HSCT) was proposed. However, the parents initially brought their child to a local pediatric hospital at two months of age due to feeding difficulties. A palatal fistula was subsequently identified, and a supportive appliance called a palatal plate was fabricated by the Oral Surgery Department of the pediatric hospital to obturate the perforation.

He underwent HSCT at the age of one year and two months, which was successful. Additionally, at one year and 10 months old, he underwent reconstructive surgery for right thumb polydactyly. Following HSCT, the fit of the palatal plate gradually worsened, and the patient was referred to our Cleft Lip and Palate Center after the polydactyly reconstruction.

**Figure 1:**
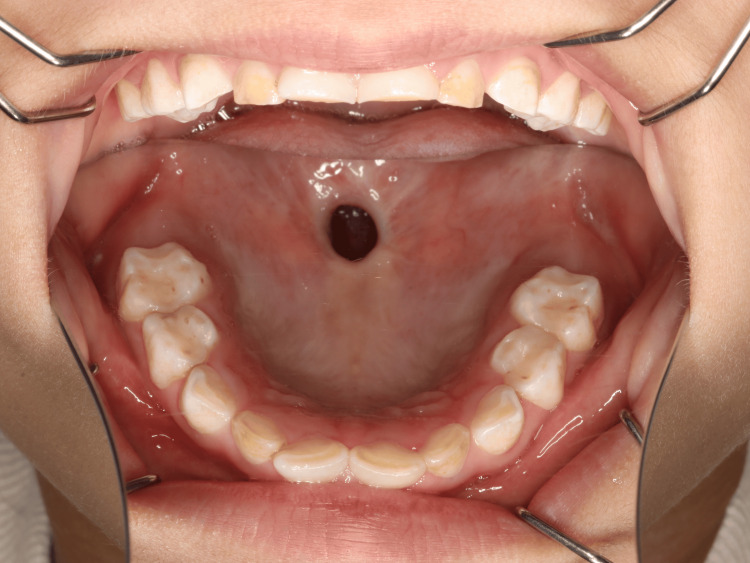
Palatal fistula A 9 × 7 mm midline fistula was observed at the junction of the hard and soft palates, with no surgical scars noted in the surrounding area.

**Figure 2:**
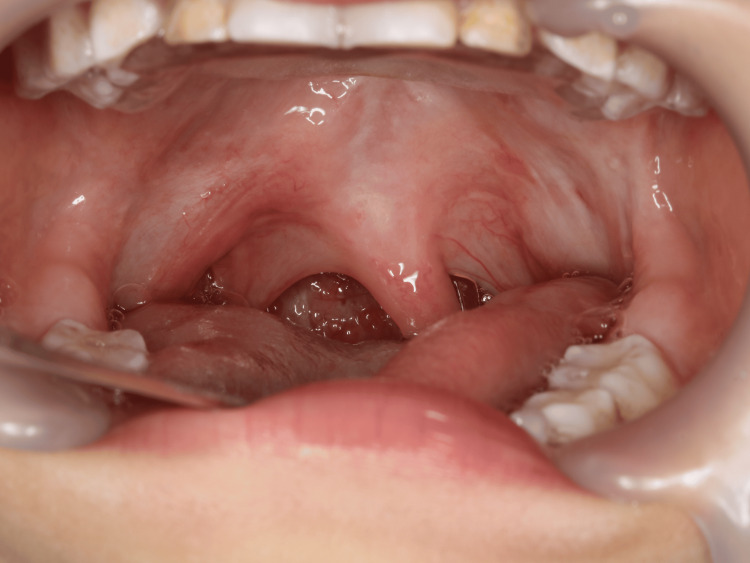
The uvula was intact, with no evidence of cleft.

**Figure 3:**
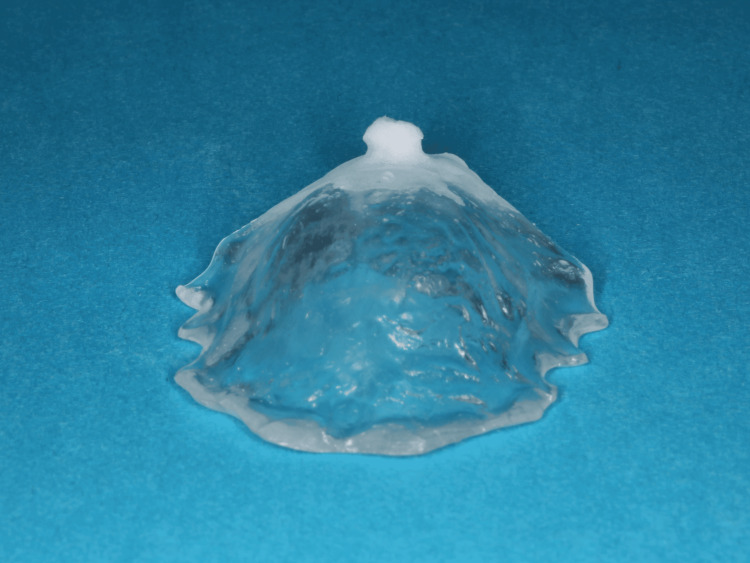
The palatal plate was fabricated from clear hard resin, with white soft resin applied around the fistula.

**Figure 4:**
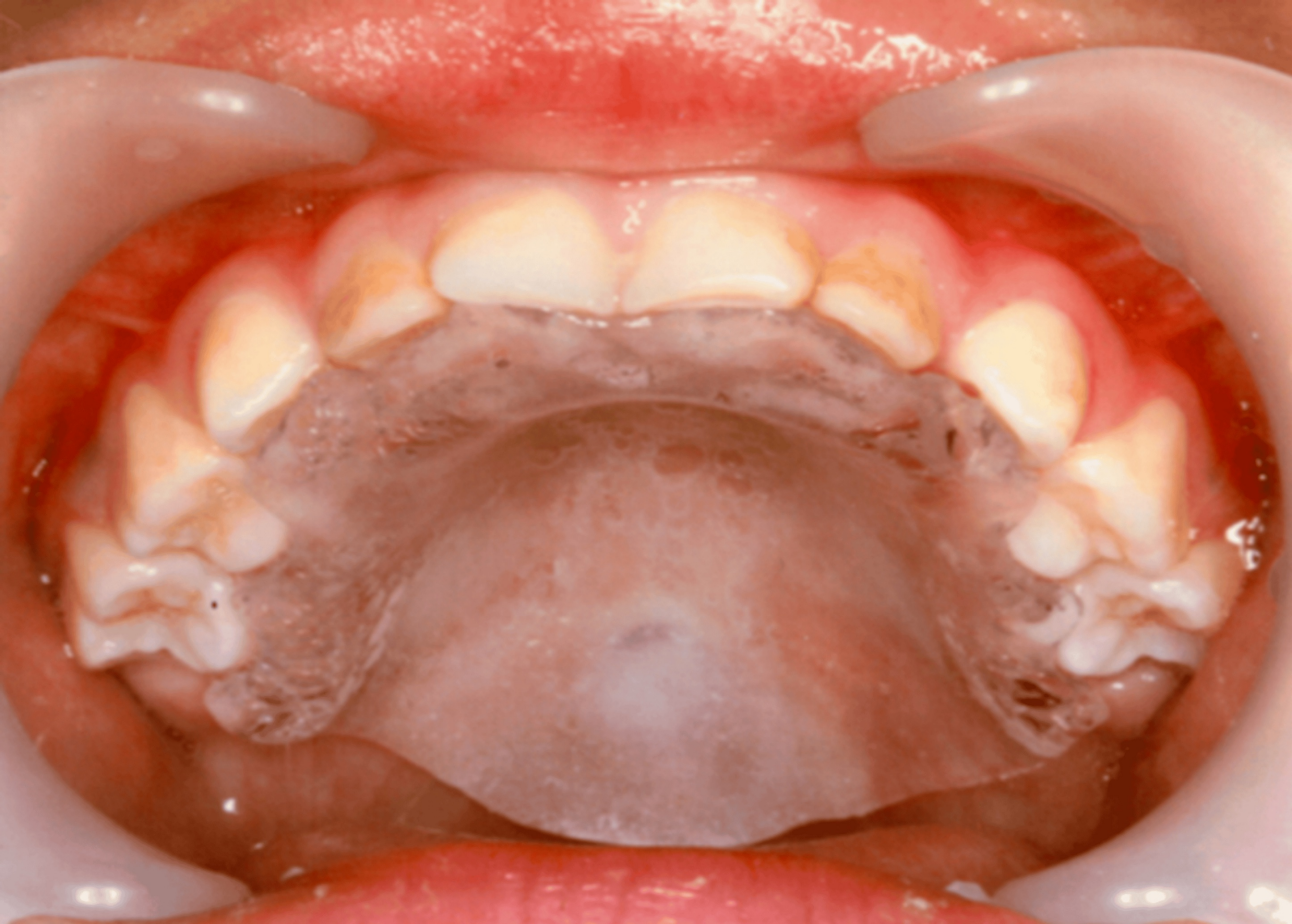
Fitting palatal fistula plate. The plate was slightly extended posteriorly to achieve closure of the fistula.

At the initial examination, a 9 × 7 mm midline fistula was observed at the junction of the hard and soft palates, anterior to the soft palate (Figure 1). The uvula appeared normal (Figure 2), and a diagnosis of SMCP was made. The patient has been on immunosuppressive therapy since undergoing HSCT. Considering the situation, the palatal impression was first conducted, and a palatal fistula plate (Figures 3-4) was fabricated to prevent nasal air emission and regurgitation leading to feeding problems. In addition, a cleft palate closure plan, which is considered for SMCP surgery, was proposed as a surgical option to be performed after completion of immunosuppressive therapy and improvement of general health. The plates were replaced as the child grew, and speech therapy was provided according to his development.

**Figure 5:**
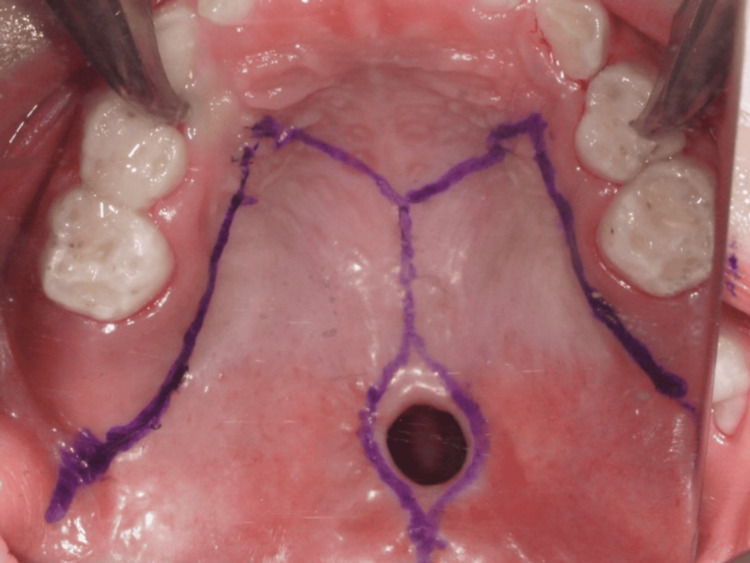
Operative finding 1: Incision design for surgical access.

**Figure 6:**
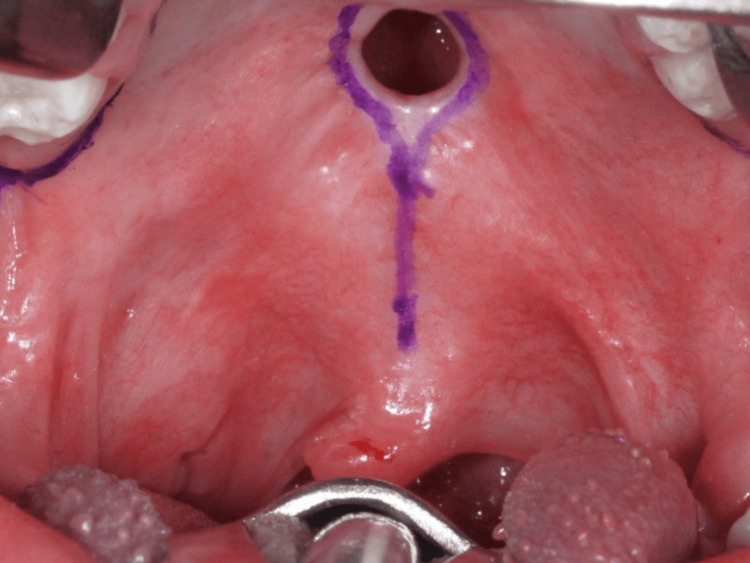
Operation finding 2: The bifid uvula was not observed; therefore, a modified Bardach technique was planned without uvula dissection.

At the age of five years, cleft palate closure surgery was performed. Preoperative blood tests showed a red blood cell count of 4.57 million/µL, white blood cell count of 3,730/µL, and platelet count of 202,000/µL, with no significant abnormalities observed. Under general anesthesia, a Bardach bilateral flap was raised using lateral, marginal, and anterior palatal incisions (Figure 5). As a result, the oral layer was gradually separated from the nasal lining. In this case, the bifid uvula was not observed; therefore, a modified Bardach technique was performed without uvula dissection (Figure 6).

**Figure 7:**
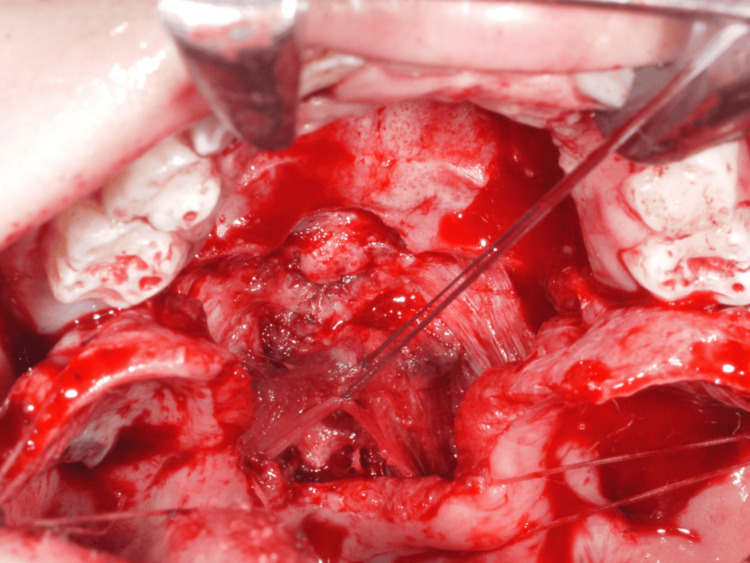
Operation finding 3: The bilateral muscle counterparts were sutured at the midline using resorbable suture material.

**Figure 8:**
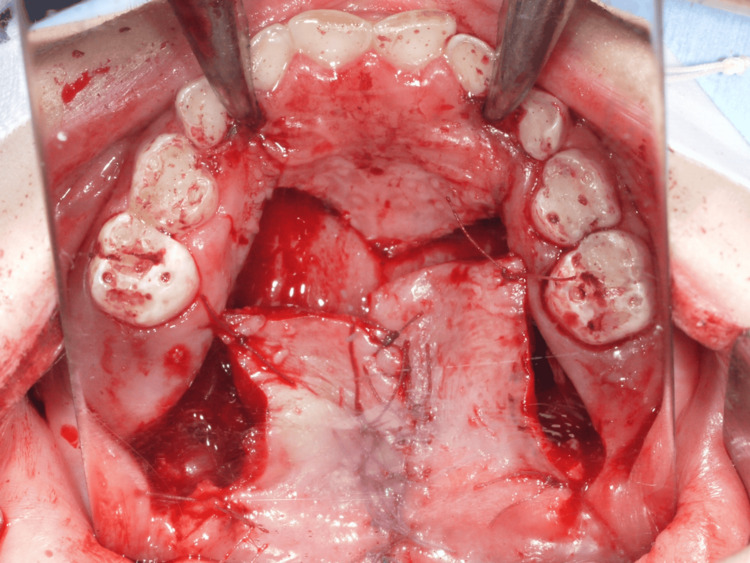
Operation finding 4. Because the oral mucosal flap was short, a pushback approach was performed to lengthen the palate.

Additionally, the palatal muscles were recognized at the specific location over the hard palate's posterior margins. The muscles were released from the medial margins; subsequently, the nasal mucosa was dissected from the soft palate muscles. The nasal layer was sutured around the fistula after mucosal dissection, and the soft palate muscles were mobilized. The bilateral muscle counterparts were then united at the midline using resorbable suture material (Figure 7). As the oral mucosal layer flaps were short, a pushback technique to lengthen the palate was considered in the treatment plan. A relatively tension-free closure was achieved using loose sutures, allowing secondary intention healing over the exposed membranous bone, which was later covered with a healing substance (Figure 8).

**Figure 9:**
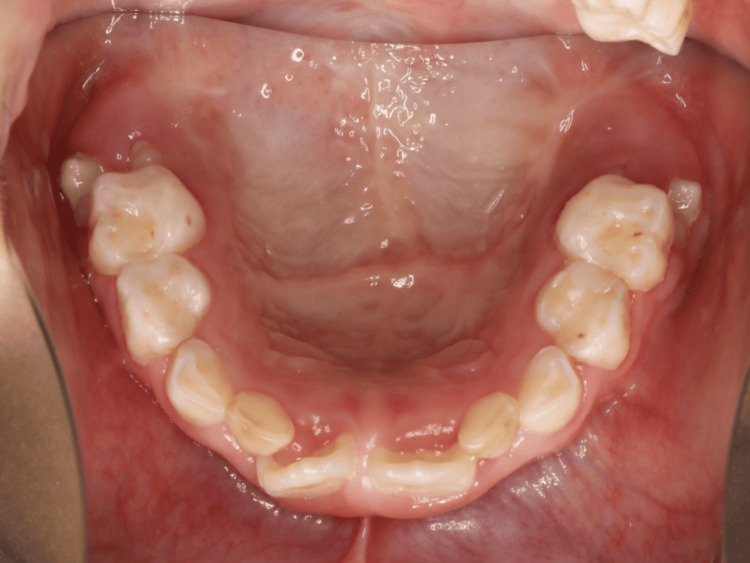
Findings six months after surgery (anterior). There was no recurrent perforation.

**Figure 10:**
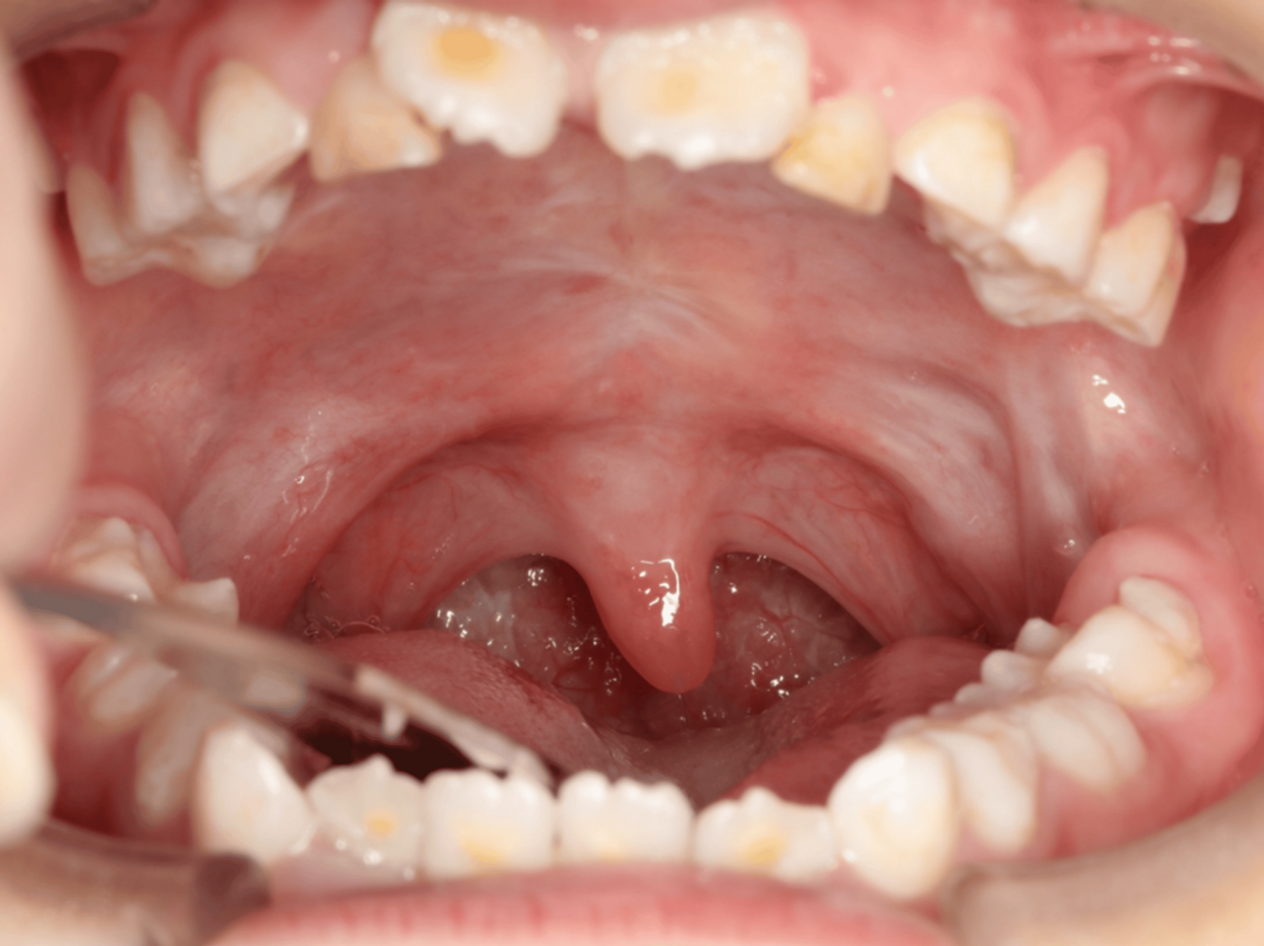
Findings six months after surgery (posterior). There was no nasal regurgitation.

The patient was under intensive postoperative care for a week and followed up for the next six months at an interval of two weeks. Before surgery, mild velopharyngeal insufficiency was observed even with the use of a palatal plate, but the functional impairment resolved after the surgery. There were no signs of complications, recurrent perforation, or nasal regurgitation (Figures 9-10).

## Discussion

Santosh et al. [8] reported that patients with WAS are highly susceptible to infections due to pre-transplantation chemotherapy and post-transplantation immunosuppression. Therefore, it is essential to thoroughly eliminate all oral sources of infection before transplantation. They also stated that special attention should be paid to fluctuations in hematological parameters during tooth extraction, and platelet transfusion should be used when necessary. Case reports of tooth extraction in patients with WAS are extremely rare [8], and to the best of our knowledge, there have been no previous reports of oral surgical procedures other than extraction. In the present case, HSCT had already been completed, and the surgery was safely performed during a period when the patient's general condition and hematological parameters were stable.

Failure of fusion of the lateral palatine processes results in a cleft palate. Embryonic development of the palate occurs between the 4th and the 12th to 13th weeks of gestation. Various causes can lead to this malformation, including: (1) defective growth of the palatal shelves, (2) failure of the shelves to elevate into the horizontal position above the tongue, (3) lack of contact between the left and right shelves (due to inadequate head extension), and (4) failure of fusion.

In this case, partial separation after palatal fusion was considered to be the cause of fistula formation. This supports the proposal by Sakuma et al. that "Rupture in the basement membrane of fused palate tissue can cause the palate to separate after fusion, which may be the cause of cleft palate" [9,10].

Although the bifid uvula was absent, the diagnosis of submucous cleft palate was made based on Calnan's triad of clinical signs. The presence of all three of Calnan’s criteria is not required for the diagnosis of SMCP. A patient may have SMCP with fewer than three of these features, or even none at all.

There are no classifications of the oronasal fistula in SMCP yet; however, Smith described a numerical fistula classification system based on anatomy. The Pittsburgh Fistula Classification System includes seven types of oronasal fistulas [11]. In this case, Type III fistulas (found at the junction of the soft and hard palates) are suitable.

HSCT is the only definitive curative treatment with tremendous outcomes for patients regarding WAS. For this reason, bone marrow transplantation performed at one year and two months of age was the most appropriate. After using immunosuppressants for a certain period, the main concern was determining the appropriate timing for surgery. With regular adjustments to the palatal obturator combined with speech therapy, concerns about excessive hypernasality and other signs of velopharyngeal insufficiency were alleviated. The patient underwent cleft surgery at five years old, after completion of immunosuppressant use and improvement in general health. In 2017, Baek et al. showed that surgical intervention for SMCP before the age of 5.5 years yields better speech outcomes [12].

After that, frequent speech-language therapy and regular follow-up may be sufficient to normalize speech. For all of that, prompt diagnosis, proposed plans, and operative interventions are vital to ensure the optimum outcomes.

This case report demonstrates the importance of dental and surgical management in a pediatric patient with WAS who underwent HSCT. WAS is a rare immunodeficiency disorder characterized by thrombocytopenia, eczema, and recurrent infections. Due to immunosuppression before and after HSCT, oral infections pose a serious risk, making pre-transplant dental evaluation and the elimination of infection sources essential.

The report also highlights the need for multidisciplinary collaboration among pediatric dentists, pediatricians, oral surgeons, and hematologists. The use of a palatal fistula plate and carefully timed surgery in consideration of the patient’s immunosuppressive condition illustrates appropriate clinical decision-making. This case serves as a useful reference for clinicians managing similarly immunocompromised patients.

## Conclusions

This case highlights several key clinical insights relevant to the management of pediatric patients with complex medical backgrounds. Early dental and craniofacial evaluation plays a crucial role in identifying palatal anomalies in children with systemic conditions such as WAS, as undiagnosed defects can lead to functional issues, including feeding difficulties and speech problems. In this case, the use of a palatal obturator provided effective temporary relief from symptoms like nasal regurgitation, demonstrating the value of prosthetic management while awaiting the appropriate time for surgery.

Surgical timing must be carefully individualized, particularly in immunocompromised patients undergoing HSCT and immunosuppressive therapy. The successful application of a modified Bardach palatoplasty, adapted to the patient’s unique anatomy and medical status, underscores the importance of surgical flexibility and precise planning. Moreover, the case reinforces the essential role of multidisciplinary collaboration among pediatricians, hematologists, oral surgeons, and speech therapists in delivering safe, comprehensive care.

Ultimately, this case contributes to the limited literature on the co-occurrence of immunodeficiency syndromes and craniofacial anomalies. It emphasizes that with early diagnosis, tailored treatment strategies, and coordinated care, favorable functional outcomes can be achieved even in medically complex patients.
